# Effect of Chinese Medicines combined with transarterial chemoembolization on primary hepatic carcinoma: A systematic review and meta-analysis

**DOI:** 10.1097/MD.0000000000034165

**Published:** 2023-06-30

**Authors:** Jianyuan Xu, Yue Shan, Chenxia Zhang, Zehua Hong, Yuanwang Qiu

**Affiliations:** a Department of Infectious Diseases, Nanjing Medical University, Nanjing, Jiangsu Province, China; b The Wuxi Fifth Affiliated Hospital of Jiangnan University, Wuxi, Jiangsu Province, China.

**Keywords:** Chinese medicines, hepatocellular carcinoma, meta-analysis, population-based study, transhepatic arterial chem otherapy and embolization

## Abstract

To systematically evaluate the survival rate and postoperative adverse reactions of patients with hepatocellular carcinoma treated with traditional Chinese medicine combined with TACE by meta-analysis.

**Methods:**

Four major literature databases (Cochrane Library, Embase, PubMed, and Web of Science) were retrieved to collect published English articles since 2009. After determining the random effect model or fixed utility model based on a heterogeneity test, odds ratios (ORs) and 95% confidence intervals (CIs) were calculated.

**Results:**

This meta-analysis included 8 prospective studies published between 2009 and 2019. Due to moderate heterogeneity (*P* < .05, *I*^2^ = 54.8%), Therefore, the random effect model is used to analyze the data, so as to explore the relationship between CMs combined with TACE treatment and survival rate and postoperative adverse reactions. All the comprehensive test results show that there is a statistical significance between CMs combined with TACE treatment and survival rate. (OR = 1.88, 95% CI 1.34–2.64, *P* = .03). Then subgroup analysis and sensitivity analysis were carried out. The results indicated that the overall results ranged from 1.12(95% CI = 1.03–1.11) to 1.21(95% CI = 1.22–1.33).

**Conclusions:**

The 1-year survival rate of patients treated with traditional Chinese medicine TACE is a protective factor, and the quality score included in the study affects the evaluation of the effective dose. At the same time, traditional Chinese medicine combined with TACE has nothing to do with the reduction of postoperative complications.

## 1. Introduction

Hepatocellular carcinoma (HCC) is the main histological subtype of primary liver cancer, accounting for 70% to 85% of primary liver cancer in the world.^[1]^ HCC is the sixth most common cancer in the world and the second leading cause of cancer death.^[2]^ It is one of the common malignant tumors in China, especially in the southeast coastal areas of China. According to cancer statistics in China, primary liver cancer is the third malignant tumor and the most common cause of death (the incidence rate is 343.7/1000 and the mortality rate is 310.6/1000).^[3]^ The main cause of hepatocellular carcinoma is cirrhosis caused by hepatitis virus infection or alcoholism. Early hepatocellular carcinoma (stage 0, stage A, and stage B) can be treated by surgery. At this stage, patients with non-cirrhosis liver have a good prognosis, but most patients with advanced hepatocellular carcinoma (stage C and D) have lost the opportunity of surgery. These patients are usually treated with transcatheter arterial chemoembolization (TACE), targeted therapy, radiotherapy and liver transplantation. Like most patients with hepatocellular carcinoma, chemoembolization (TACE) is the most commonly used palliative treatment for patients with unresectable hepatocellular carcinoma, because it can improve the survival of patients.

TACE is a minimally invasive operation in interventional radiology, which is used to limit the blood supply of tumor. It combines chemotherapy with embolization. Although this kind of surgery can rarely cure liver cancer, research shows that in 70% or more patients, the cancer shrinks and patients may live longer.^[4–6]^There are still some risks and shortcomings in TACE, such as the risk of infection after embolization, the embolism staying in the wrong position and losing the blood supply of normal tissues, the contrast agent allergic reaction, kidney damage and the reaction to chemotherapy, which may include nausea, alopecia, leukopenia, thrombocytopenia and anemia.^[4,7]^

Chinese medicines (CMs) are commonly used in the treatment of HCC, but its side effects are rarely reported. More and more studies have been done to evaluate the influence of CMS on HCC. Studies have confirmed that the chemoprevention and anti-hepatoma properties of CMS are mainly realized by inducing apoptosis and autophagy of cancer cells and cytotoxicity to cancer cells.^[8–10]^

## 2. Materials and methods

### 2.1. Literature retrieval

By searching 4 major literature databases (Cochrane Library, Embase, PubMed, and Web of Science), the open published clinical trials compared the clinical efficacy of Chinese herbal medicine plus TACE versus TACE only in patients with advanced hepatocellular carcinoma were collected.

The searching strategy was performed using “hepatocellular carcinoma,” “liver carcinoma,” “hepatocellular carcinoma/cancer” AND “Medicine, Chinese Traditional,” as the Medical Subject Headings (MeSH) and corresponding free text word searching term. The title and abstract of initial identified articles were evaluated for appropriateness to the inclusion criteria. To prevent research from being missed, the references in the studies retrieved from the online databases and previously published systematic reviews were also manually searched to further identify relevant studies.

### 2.2. Study selection

The inclusion criteria of this meta-analysis were: published as an original article; belonged to clinical trial, Including prospective studies, not limited to randomized clinical trials, controlled clinical trial, prospective study; The intervention measures of the treatment group were Chinese medicine combined with TACE, and the control group was TACE alone; provided the number of participants; and The risk estimates were reported with corresponding 95% confidence intervals (95% CIs). Meanwhile, studies were excluded with one or more of the following criteria: review articles; animal trials; conference papers; data unavailable to be extracted; and The treatment group was treated with traditional Chinese medicine combined with other drugs; The control group received TACE combined with other drugs.

### 2.3. Data extraction and quality assessment

The full text of all articles included was reviewed. Data abstraction and full-text review were carried out independently by 2 authors, and discrepancies were corrected by the third. To remove duplicates, we imported the extracted study into the Endnote Software X9.0, followed by the screening of titles and abstracts by 2 authors. And the MOOSE (meta-analysis of observational studies in epidemiology) guidelines were followed.^[11]^ Data collection was conducted using standardized forms developed by the research team. Data extraction included the following information: study characteristics, such as types, authors, year of publication, number of patients and sample size. An analysis of clinical indicators and data was conducted: case/participants; 1-year survival number; Intervention measures of treatment group; Child-Pugh classification; year; and age. The study quality assessment was performed following the Newcastle-Ottawa Scale.^[12]^ The scoring system assessed 3 aspects of a study: selections (representativeness of cohort and exposure assessment); comparability (confounding determination) and outcomes (assessment of the outcome and follow-up). The studies were rated based on selection, comparability, exposure, and outcome, and scored with a maximum of 6 points. There were 2 categories of papers: high-quality (study score ≥ 6) and low-quality (study score < 6).^[13,14]^

### 2.4. Statistical analysis

The meta-analyses were performed using Stata14.0. Since the indices collected in this study were dichotomous variables, the odds ratio (OR) was used as the effect size in the statistical analysis. The *I*^2^ index and Cochran’s Q tests were employed to quantify incoherence and heterogeneity between studies, respectively. *I*^2^ was evaluated as a measure of heterogeneity across studies, which was interpreted as not significant (0–40%), moderate heterogeneity (30–60%), substantial heterogeneity (50–90%), or large heterogeneity (75–100%).^[15]^ If no statistical heterogeneity existed in pooled studies (*P* > .1, *I*^2^ ≤ 50%), we adopted a fixed-effect model for Meta-analysis, otherwise, a random-effect model was applied (*P* < .1, *I*^2^ > 50%). A sensitivity and subgroup analysis were performed to explore potential causes of heterogeneity. There were several confounding factors, including quality scores, Intervention measures, and study types. We assessed the sources of heterogeneity by analyzing the previously described factors in the subgroups. Meanwhile, analyses of sensitivity were conducted to evaluate the robustness of the main outcomes. Furthermore, Egger’s correlation tests regressed the publication bias, the *P* value at .05 was considered statistically significant, and the test results were attached to the paper.^[16]^

### 2.5. Ethics of ethics

All analyses were based on previous published studies, thus no ethical approval and patient consent are required.

## 3. Results

### 3.1. Search results

**Figure 1.**
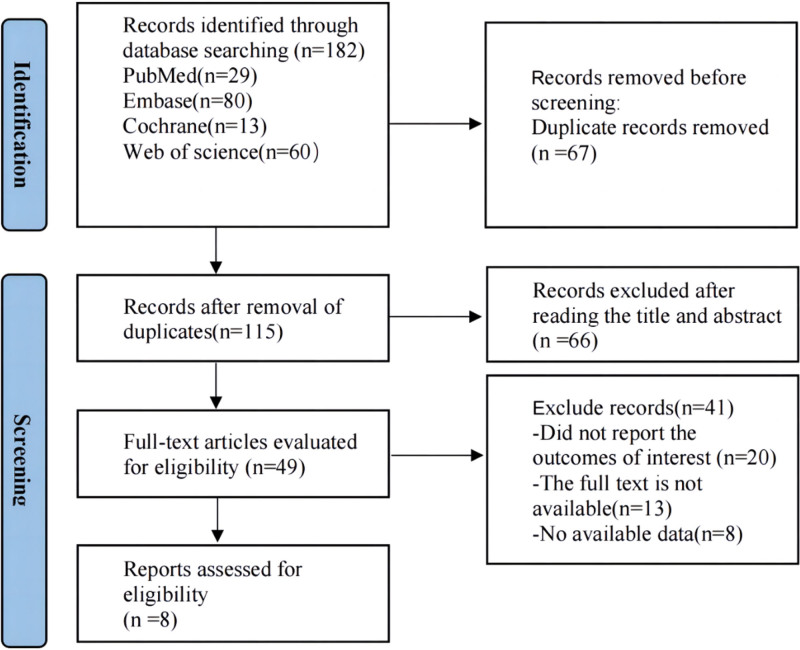
Description of the entire process from literature retrieval to the selection of 8 target articles.

A total of 182 relevant studies were retrieved from the initial literature review. Duplicate articles were firstly removed among predefined databases based solely on titles. Additionally, to 67 duplicate articles, the remaining 115 studies were also screened by reviewing titles and abstracts. In addition, 66 studies based on animals, review articles, and case reports were also ruled out from this work. A comprehensive review of 49 studies was conducted. Twenty articles were excluded due to missing results of interest, 13 articles were ruled out because of inaccessible full texts, and 8 with data unavailable. Ultimately 8 articles were included for meta-analysis.^[17–24]^ The process of literature retrieval was shown in Figure 1.

### 3.2. Study characteristics, and quality assessment

A summary of the characteristics of the included studies were presented in Table 2. The included 8 studies were published between 2009 and 2019, which included total 1013 participants. These studies were conducted in China. There are 4 studies with quality evaluation over 6 points and less than 6 points respectively. In addition, 4 were case-control studies, 1 were cohort studies, and 3 was a randomized controlled trial. Traditional Chinese medicine combined with TACE is related to the improvement of 1-year survival rate in all studies.

As shown in Table 1, the NOS scores for all included studies ranged from 4 to 6 points. Five in 8 studies were high quality. In terms of selection and outcome bias, all studies conformed to the inclusion criteria.

**Table 1:** The general characteristics of included trials.

Author	Year	Study type	Events	Country	Age	NOS	Intervention measure
GuangSheng Zhao	2017	Randomized controlled trials	54	Chinese	36–79	6	TACE combined with Huaier granule
Hetong Zhao	2017	Case-control study	142	Chinese	55–67	5	TACE combined with Jie-du granule
JingHao Zhang	2019	Randomized controlled trials	108	Chinese	18–65	6	TACE combined with Ganji Formulation
Yang Yu	2009	Case-control study	165	Chinese	55–66	5	JDF granule preparation (a traditional Chinese herbal medicine formula)
ChengWu Tang	2016	Randomized controlled trials	103	Chinese	46–58	6	TACE combined with Jianpi ligan decoction
Hua Xu	2019	Case-control study	125	Chinese	18–70	5	TACE combined with Chaihu-huaji decoction
YueMeng Wan	2018	Prospective cohort study	89	Chinese	18–75	6	TACE combined with Kang’ai Injection
LingLing Sun	2018	Case-control study	227	Chinese	20–83	6	TACE combined with Chinese herbal medicine

TACE = transhepatic Arterial Chem Otherapy and Embolization.

## 4. Chinese medicine combined with TACE and 1 year survival rate

### 4.1. Heterogeneity test

**Figure 2.**
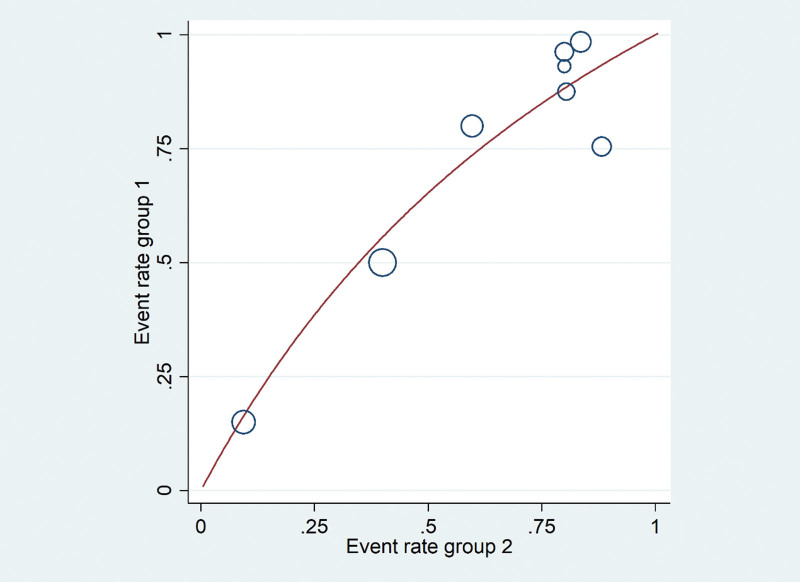
By Labbe plot, it was confirmed that there was mild to moderate heterogeneity among the literatures in this study.

Eight papers in this study have been tested for heterogeneity, *I*^2^ = 54.8%, and *P* = .03 < 0.05 in Q test, which indicates that there is slight to moderate heterogeneity among the papers in this study. Further investigation of Labbe diagram indicates that some papers may have strong heterogeneity, and the results are shown in the Figure 2:

According to the analysis in Figure 2, it is concluded that there is slight to moderate heterogeneity among the literatures in this study, so we can also combine the effect quantities through the fixed effect model.

### 4.2. Fixed effect combined effect quantity

**Figure 3.**
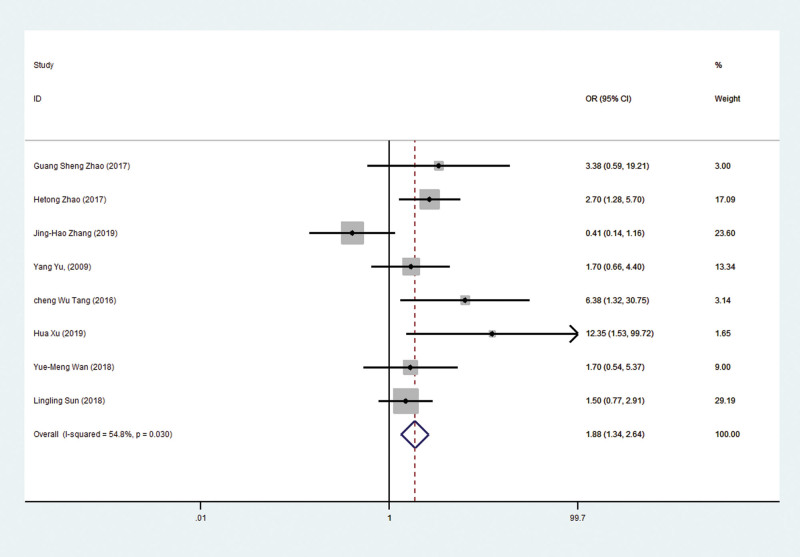
A fixed-effect model was used to analyze the ORs of 8 articles to compare the association between the 1-year survival rate of patients with HCC and the treatment of traditional Chinese medicine combined with TACE. OR = 1.88, 95% CI 1.34–2.64, *P* = .03. CIs = confidence intervals, HCC = hepatocellular carcinoma, ORs = odds ratios, TACE = transhepatic Arterial Chem Otherapy and Embolization.

As shown in Figure 3, the fixed effect model combined with OR was selected, and OR = 1.88 (1.32–2.64), which means that the 1-year survival rate of liver cancer patients treated with traditional Chinese medicine combined with TACE is 1.88 times higher than that treated with TACE alone, with statistical significance (*Z* = 3.64, *P* = .03 < .05).

### 4.3. CMs combined with TACE treatment on survival and other clinical outcomes in HCC patients

This study further explored the correlation between the combination of traditional Chinese medicine and TACE and the survival rate of patients at 6 months, 2 years, 3 years, and the adverse reactions of patients after TACE alone. Due to mild heterogeneity between studies, a fixed effects model was used. It was found that there was no statistically significant correlation between the 6-month (7 studies), 2-year (8 studies), and 3-year survival rate (7 studies) of HCC patients treated with traditional Chinese medicine combined with TACE. At the same time, traditional Chinese medicine combined with TACE cannot relieve the fever, leukopenia, nausea, diarrhea, vomiting, fatigue, loss of appetite and stomach upset after TACE. The detailed results are shown in the following table.

### 4.4. Sensitivity analysis

**Figure 4.**
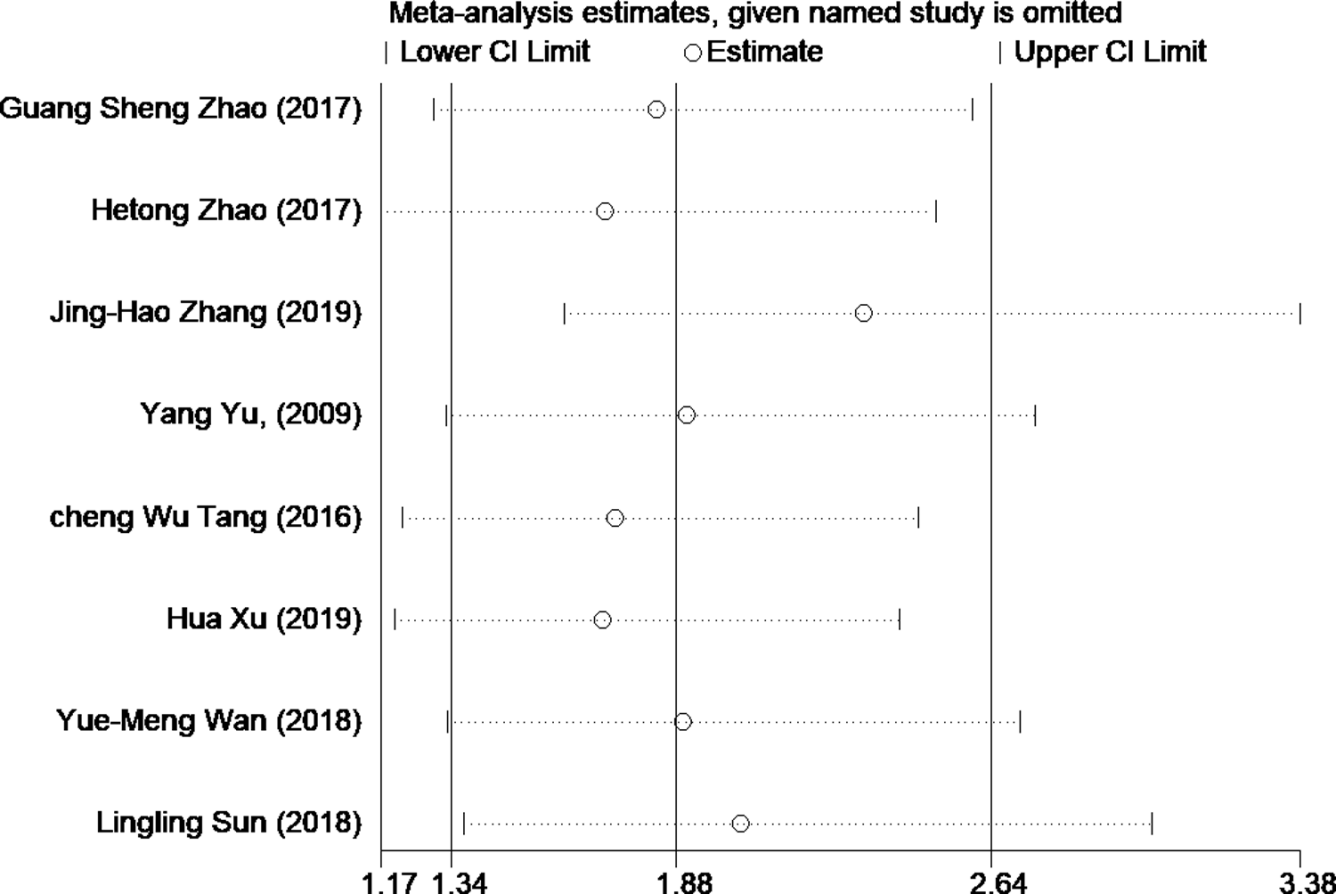
Sensitivity analyses were performed to investigate potential sources of heterogeneity and showed the main result was robustness. The overall results ranged from 1.12 (95% CI = 1.03–1.11) to 1.2 1(95% CI = 1.22–1.33). CIs = confidence intervals.

Potential sources of heterogeneity were investigated using a sensitivity analysis. Following the exclusion of one study, we analyzed the other studies. The results of the sensitivity analysis were shown in Figure 4. Excluding any single study, the overall results ranged from 1.12 (95% CI = 1.03–1.11) to 1.21 (95% CI = 1.22–1.33), implying that the main results were robust.

### 4.5. Subgroup analysis

Moderate heterogeneity was detected among these 8 trials (*P* = .03, *I*^2^ =54.8%). Subgroup analyses were performed based on Intervention measures, quality score, study type, and year of publication (Table 2). These subgroups all have moderate statistical differences.

**Table 2:** Subgroup analysis of Chinese medicine combined with TACE treatment and 1-year survival rate of HCC patients.

Group	Studies (n)	OR (95% CI)	*P*	Heterogeneity test	
				*P*	*I*^2^ (%)
Total	8	1.88 (1.34–2.64)	.030	.030	54.3
Design					
Case-control	4	1.134 (0.990–1.298)	0	.218	32.4
Cohort	1	0.638 (0.363–1.122)	.368	NA	NA
Randomized controlled trials	3	1.631 (1.230–2.162)	.396	.007	79.6
Intervening measure					
Single	5	2.60 (1.62–4.18)	0	.473	0
Mixed	3	1.31 (0.80–2.15)	.279	.012	77.3
Publication year					
<2018	4	0.588 (0.321–1.075)	0	.548	0
≥2018	4	0.894 (0.473–1.690)	.145	.022	68.9
Quality score					
<6	4	0.94 (0.94–2.25)	.092	.04	60.1
≥6	4	2.78 (1.60–4.82)	0	.224	33.2

CIs = confidence intervals, HCC = hepatocellular carcinoma, ORs = odds ratios, TACE = transhepatic Arterial Chem Otherapy and Embolization.

In subgroup stratified by Intervention measures, data from single medicine subgroup analysis suggested that single Chinese medicine combined with TACE is effective for 1-year survival rate (OR = 2.60, 95% CI 1.62–4.18, *P* = 0 < .01). Conversely, data from the Mixed Chinese medicine treatment combined with TACE subgroup (OR = 1.31, 95% CI 0.80–2.15, *P* = .279 > .05) exhibited opposite results. It is worth noting that, in subgroups of these 4 studies with a quality score of more than 6 (OR = 2.78, 95% CI 1.6–4.82, *P* = 0 < .01), the combination therapy was associated with the prolongation of the 1-year survival rate of patients, which was considered as a protective factor.

### 4.6. Bias test

**Figure 5.**
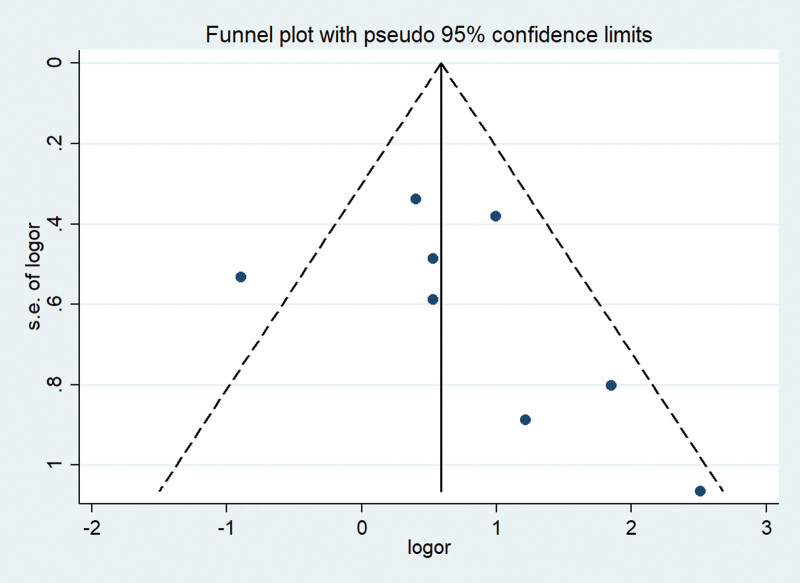
The funnel plot test confirmed that there was no significant publication bias between the 1-year survival rate of HCC patients and the combination of CMs and TACE treatment. CMs = Chinese medicines, HCC = hepatocellular carcinoma, TACE = transhepatic Arterial Chem Otherapy and Embolization.

As shown in the Figure 5, the funnel chart bias analysis results show that the scattered points of funnel chart are mostly symmetrical, and there is an obvious deviation, which may lead to publication bias.

### 4.7. Publication bias

**Figure 6.**
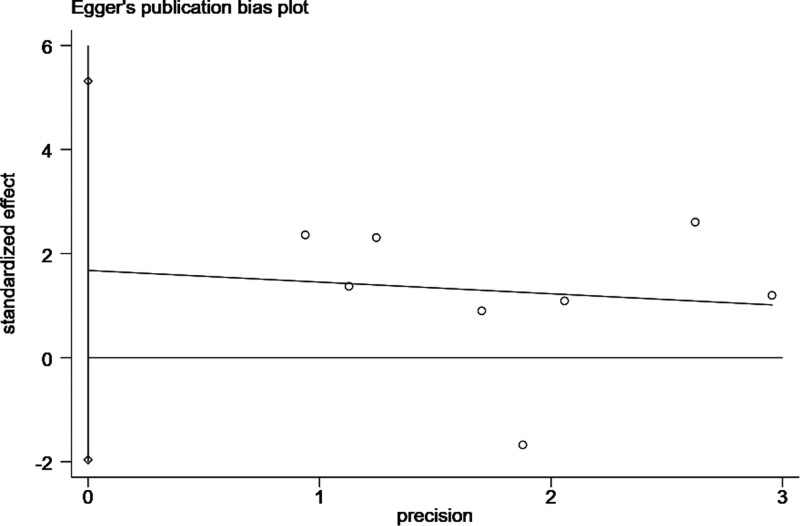
Egger test and Egger test plot were performed to confirm that there was no significant publication bias between the 1-year survival rate of patients with HCC and the treatment of traditional Chinese medicine combined with TACE. *P* = .303. HCC = hepatocellular carcinoma, TACE = transhepatic Arterial Chem Otherapy and Embolization.

To detect publication bias in the included studies, Egger tests were conducted and the findings were visualized as well (Fig. 6). Data showed that there was no significant publication bias between tea consumption and CRC risk (*P* = .303) by Egger’s tests.

## 5. Discussion

Primary HCC or HCC is the sixth most common malignant tumor in the world, with 626,000 cases and the third most common cause of cancer death (598,000 deaths per year).^[25]^At present, there are many treatments for liver cancer, but due to the lack of effective early screening methods, 70% to 80% of patients are diagnosed at the middle and late stage, resulting in a 5-year overall survival rate of liver cancer of only 14.1%.^[3,26]^Hepatocellular carcinoma is a multi-factor and multi-stage process, so it is difficult to find a specific drug. Therefore, the combination therapy is most likely to be a new strategy for the treatment of liver cancer in the future, which has reached a broad consensus in academic circles.^[27]^Marelli et al reviewed the research on combined therapy strategies and found that TACE combined with percutaneous ablation, percutaneous ethanol injection or radiofrequency ablation can improve the survival rate.^[27]^

In this study, we comprehensively evaluated the effect of traditional Chinese medicine combined with TACE on the survival rate of primary liver cancer compared with TACE alone through meta-analysis, and found that from the current data, the combined treatment prolonged the 1-year survival rate of patients. (OR = 1.88, 95% CI 1.339–2.642). However, there was no significant difference in the 6-month, 2-year, and 3-year survival rates between this combination therapy and TACE alone. Similarly, the combination therapy did not improve the success rate of patients. Whether the combined treatment can alleviate the postoperative complications of patients, the research shows that there is no obvious difference in postoperative leukopenia, nausea, fever, diarrhea, vomiting, fatigue, loss of appetite and stomach upset (Tables 3 and 4).

**Table 3:** The 6 months, 1 year, 2 years and 3 years survival rate of patients with HCC treated with traditional Chinese medicine combined with TACE.

Index	Studies	Sample size	Heterogeneity test	Analytical model	OR	95% CI	*P*
6 month survival	7	918	*P* = .053, *I*^2^ = 54.1%	Random (M-H heterogeneity)	1.88	1.219–2.901	.004
1 year survival	8	1013	*P* = .030, *I*^2^ = 54.8%	Fixed Mantel-Haenzel	1.88	1.339–2.642	.053
2 year survival	8	1021	*P* = .141, *I*^2^ = 36.1%	Fixed Mantel-Haenzel	1.983	1.483–2.651	.141
3 year survival	7	794	*P* = .411, *I*^2^ = 1.8%	Fixed Mantel-Haenzel	3.218	2.148–4.820	.411

CIs = confidence intervals, HCC = hepatocellular carcinoma, ORs = odds ratios, TACE = transhepatic Arterial Chem Otherapy and Embolization.

**Table 4:** Chinese medicine combined with TACE in the treatment of postoperative complications in patients with HCC.

Index	Studies	Sample size	Heterogeneity test	Analytical model	OR	95% CI	*P*
Treatment success rate	4	398	*P* = .568, *I*^2^ = 0%	Fixed Mantel-Haenzel	4.428	2.227–8.806	0
Fever	4	426	*P* = .268, *I*^2^ = 23.9%	Fixed Mantel-Haenzel	0.7	0.436–1.124	0.14
Leukocytopenia	2	187	*P* = .994, *I*^2^ = 0%	Fixed Mantel-Haenzel	0.56	0.220–1.422	0.223
Nausea	3	323	*P* = .460, *I*^2^ = 0%	Fixed Mantel-Haenzel	0.548	0.279–1.076	0.08
Diarrhea	2	170	*P* = .846, *I*^2^ = 0%	Fixed Mantel-Haenzel	1.289	0.516–3.217	0.587
Vomit	2	215	*P* = .532, *I*^2^ = 0%	Fixed Mantel-Haenzel	0.379	0.156–0.924	0.033
Fatigue	4	363	*P* = .186, *I*^2^ = 37.7%	Fixed Mantel-Haenzel	0.638	0.371–1.099	0.105
Anorexia	4	380	*P* = .065, *I*^2^ = 58.4%	Random (M-H heterogeneity)	0.64	0.381–1.074	0.091
Stomach upset	3	273	*P* = .595, *I*^2^ = 0%	Fixed Mantel-Haenzel	1.165	0.617–2.201	0.637

CIs = confidence intervals, HCC = hepatocellular carcinoma, ORs = odds ratios, TACE = transhepatic Arterial Chem Otherapy and Embolization.

Wang think that Aidi injection combined with TACE can improve the 1-year survival rate of patients.^[28]^Dong and Meng also found similar results.^[28]^However, Li, Yang, Dong, Xue, etc. think that the combination therapy has no significant difference in prolonging the 1-year survival rate.^[29]^This meta-analysis in 2014 showed that cinobufotalin combined with TACE could significantly improve the objective effective rate and 2-year survival rate compared with TACE alone. In meta published in 2017, a comprehensive report summarized the research progress of anti-tumor mechanism of aidi injection. In vivo experiments showed that Aidi injection had obvious inhibitory effect on S180 and H22 solid tumors in mice and could enhance the nonspecific and specific immune function of mice.^[28]^

Similarly, in this study, it was found that the 1-year survival rate of patients with single medicine combined with TACE was significantly prolonged among patients with single medicine and mixed Chinese medicine preparations, with OR = 2.60, 95% CI (1.62–4.18), *P* = 0 < .01. On the contrary, mixed medicine combined with TACE had no such effect. This result may be caused by the interaction between mixed preparations. In these 4 studies with a quality score of more than 6, the combination therapy was associated with the prolongation of the 1-year survival rate of patients, which was considered as a protective factor. The possible reason is that the higher the quality of articles, the more rigorous the experimental design.

This analysis recruited 1013 participants. All relevant prospective studies (n = 8) were included in this study, and the risk of publication bias was low (*P* = .303 > .05); And through sensitivity analysis, it is confirmed that there is no significant heterogeneity or deviation; In addition, the difference of short-term (adverse reaction after TACE) and long-term (survival) effectiveness between traditional Chinese medicine combined with TACE and TACE alone was shown by forest diagram. It must be said that there are still some limitations in this study. First, the current meta-analysis fails to eliminate heterogeneity, whether in overall analysis or subgroup analysis. Second, it was found in this review that the components of CMs prescriptions vary significantly, which may be due to the differences in CMs diagnosis and personal experience in CMs. The third point is that it is currently uncertain whether this combination modality will cause new adverse reactions and/or increase the financial burden on patients because these issues have not been reported in the included literature. Finally, due to the relatively small sample size of some included articles, the statistical strength may be limited, and it is difficult to generalize the results.

However, this meta-analysis needs to consider several limiting factors. First, the overall quality of the included individual studies is relatively low; Second, the average number of subjects enrolled in each study was small, and no multicenter randomized controlled trial was included in this meta-analysis. Third, survival data for each study were incomplete, resulting in inability to calculate risk ratios.

To sum up, this meta-analysis shows that traditional Chinese medicine combined with TACE is a protective factor for prolonging the 1-year survival rate of patients (OR = 1.88, 95% CI 1.34–2.64). These relationships need to be confirmed by more well-designed large-scale prospective studies and randomized clinical trials.

## 6. Conclusion

TACE combined with traditional Chinese medicine is a protective factor for prolonging the 1-year survival rate of patients. Compared with mixed Chinese medicine decoction, single medicine is more meaningful for prolonging the survival rate of patients. Combination of TACE with traditional Chinese medicine cannot effectively relieve the postoperative adverse reactions of patients.

## Acknowledgments

We appreciate the guidance and support from YWQ on this study.

## Author contributions

**Conceptualization:** Chenxia Zhang.

**Data curation:** Yue Shan.

**Methodology:** Yue Shan.

**Software:** Zehua Hong.

**Supervision:** Yuanwang Qiu.

**Writing – original draft:** Jianyuan Xu.

**Writing – review & editing:** Jianyuan Xu.
